# Supplementary material for: Hydrothermal Stability of Hydrogen-Selective Carbon–Ceramic Membranes Derived from Polybenzoxazine-Modified Silica–Zirconia
Source: Membranes (Basel). 2022 Dec 26;13(1):30. doi: 10.3390/membranes13010030 (PMC9860565; doi:10.3390/membranes13010030)
Supplement: Supplementary file 1 [file membranes-13-00030-s001.zip › membranes-2079069-supplementary.pdf]

membranes

Supplementary Materias

Hydrothermal Stability of Hydrogen-Selective Carbon–Ceramic Membranes Derived from Polybenzoxazine-Modified Silica–Zirconia

Sulaiman Oladipo Lawal, Hiroki Nagasawa, Toshinori Tsuru and Masakoto Kanezashi *

Chemical Engineering Program, Graduate School of Advanced Science and Engineering, Hiroshima University, 1-4-1 Kagamiyama, Higashi-Hiroshima 739-8527, Japan
* Correspondence: kanezashi@hiroshima-u.ac.jp

Table S1. A comparison of popular H_2 separation technologies.

Technology	Working Principle	Purity of Produced H_2 [%]	Advantages	Disadvantages
Pressure swing adsorption	Adsorption-desorption cycles of target/impurity gases	99.99 (from SMR) [S1]	Mature and commercially available<br>No external heating required	Expensive<br>Complicated operation procedures
Cryogenic distillation	Utilizes the difference in boiling temperatures of the gas mixture components	95 or less [S2]	Mature and commercially available<br>Low-temperature process	Expensive<br>Energy intensive<br>Very high H_2 purity not practical
Membrane separation	Thin barriers allow only the permeation of selected molecules through them	99.999 (via Pd-based dense metal membranes) [S3]	Mature and commercially available<br>Ease of operation<br>Low cost [S4]<br>Low energy consumption [S4]<br>Can be integrated into as chemical reactors	Less mechanically durable<br>Stability in chemical and hydrothermal applications

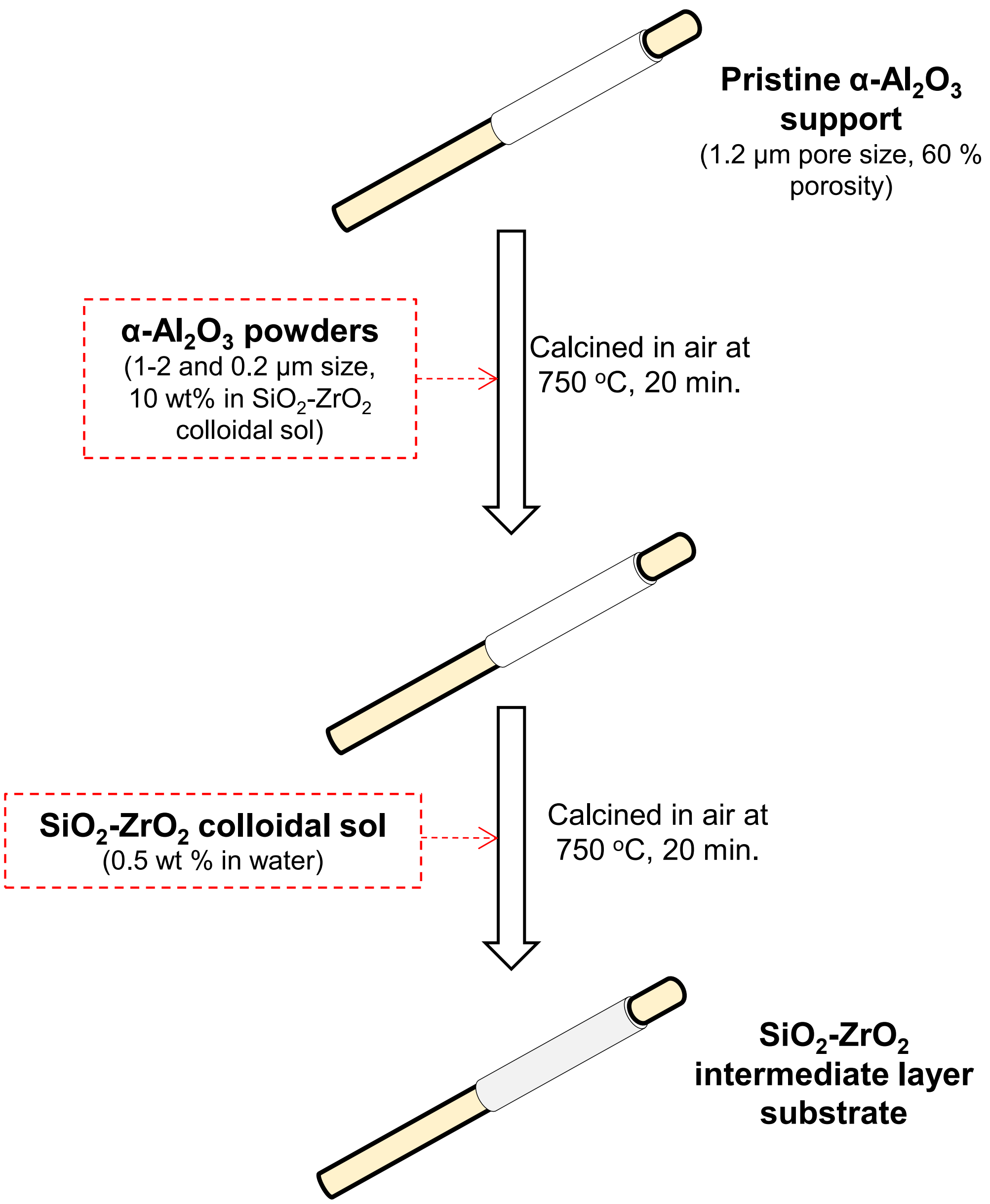

Figure S1. Flow of membrane support fabrication.

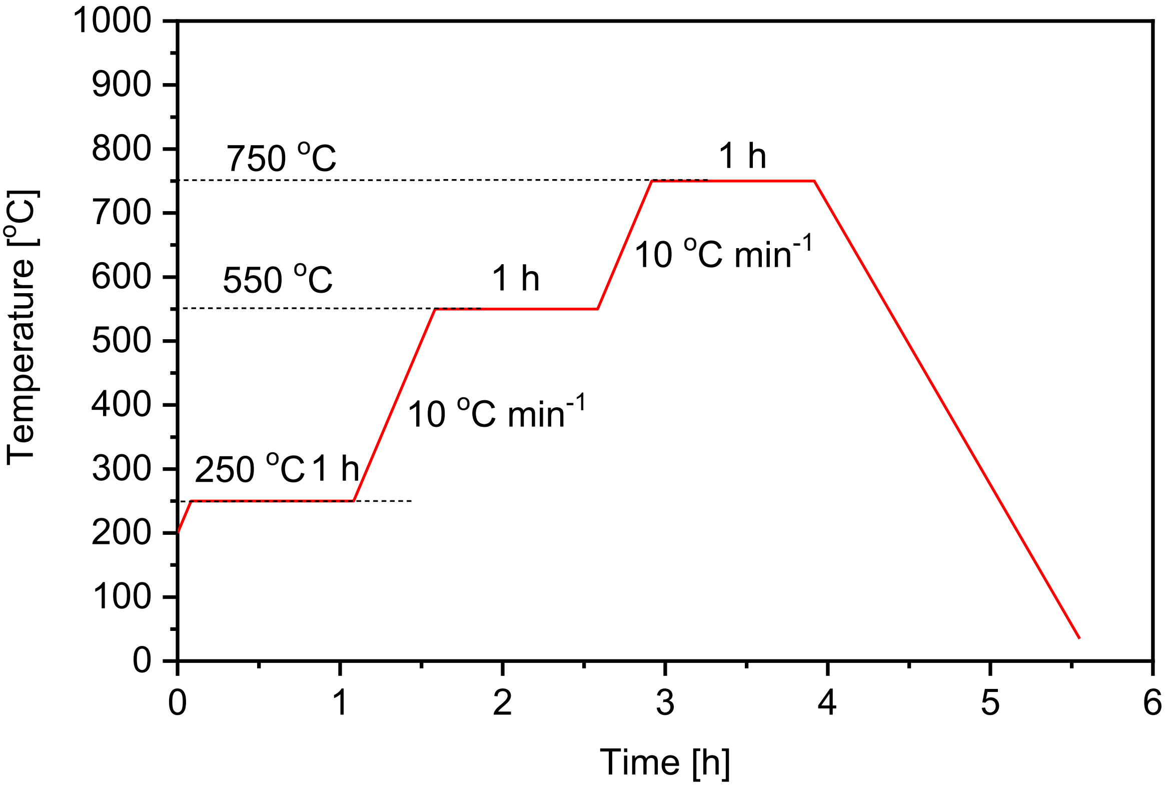

Figure S2. C-SZ membrane pyrolysis sequence.

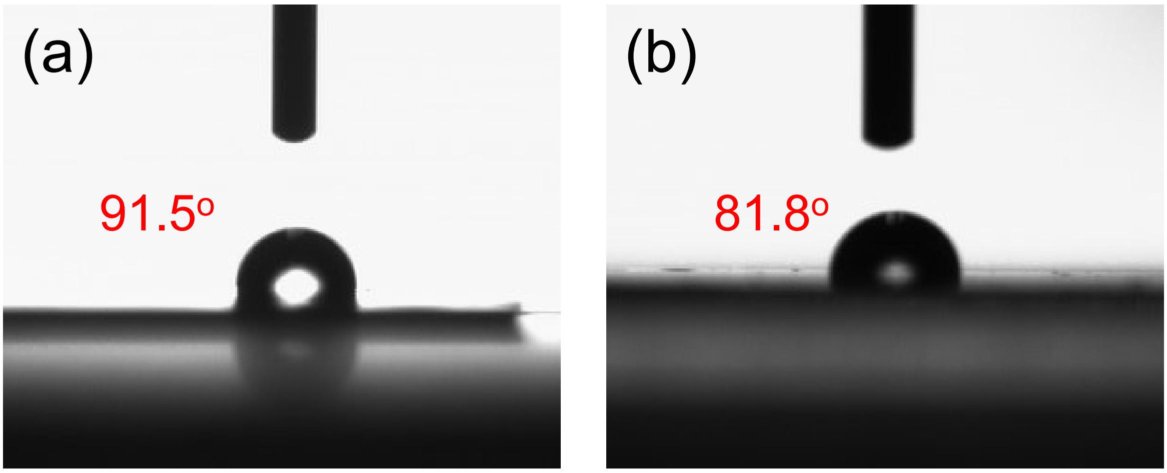

Figure S3. Water-contact angles of (**a**) fresh and (**b**) 90 °C-cured SiO_2-ZrO_2-polybenzoxzine films.

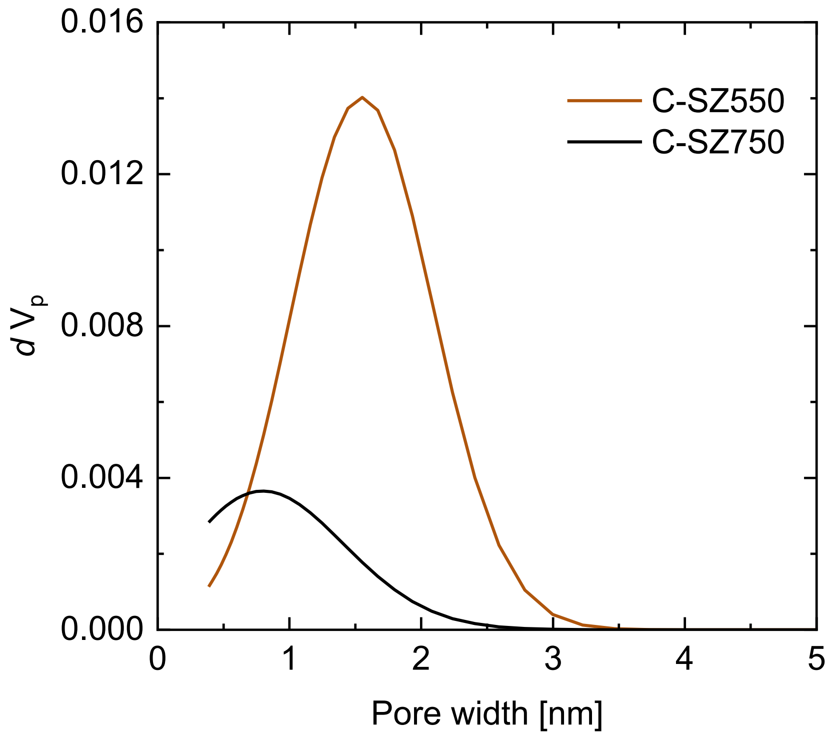

Figure S4. Non-local density functional theory (NLDFT)-derived pore size distribution for carbon-SiO_2-ZrO_2 powders prepared at 550 (C-SZ550) and 750 °C (C-SZ750).

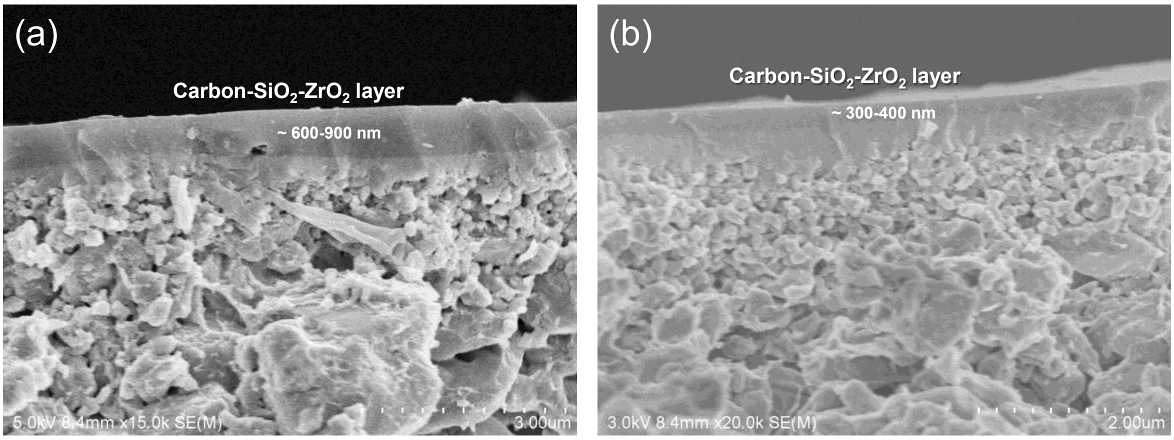

Figure S5. Scanning electron microscopy images of the cross-sections of carbon-SiO_2-ZrO_2 membranes prepared at (**a**) 550 and (**b**) 750 °C.

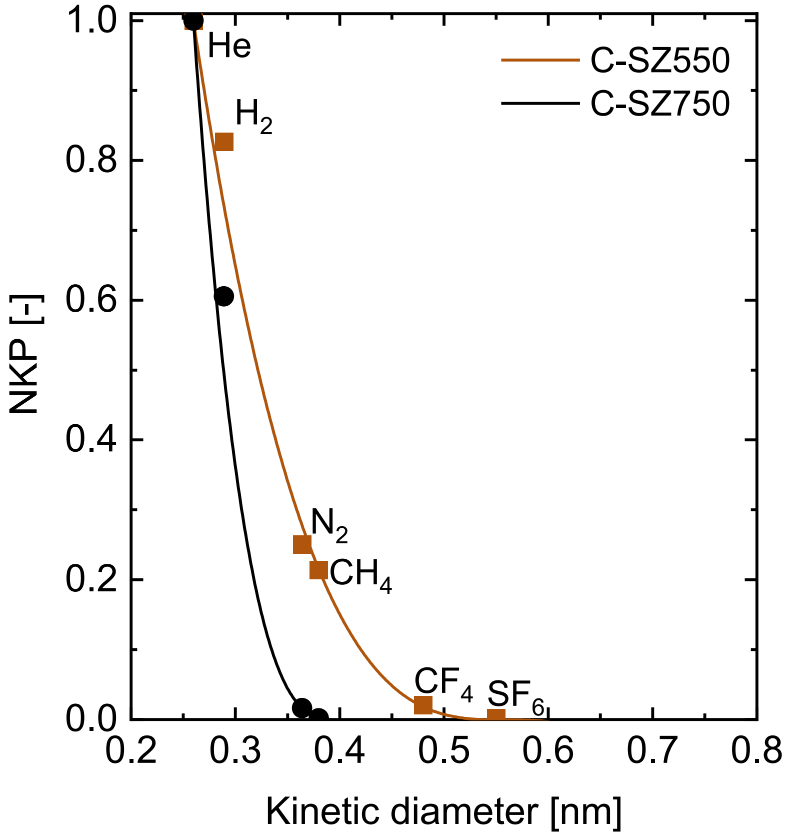

Figure S6. Pore size distributions of C-SZ550 and C-SZ750 membranes determined by normalized Knudsen permeance as a function of kinetic diameter of gases.

Table S2. Membrane performance characteristic values measured at 300 °C before (0 kPa) and after (90 and 150 kPa) steam treatment at 500 °C.

Steam condition [kPa]	C-SZ550 membrane			C-SZ750 membrane		
	H_2 permeance [10^{-7} mol m^{-2} s^{-1} Pa^{-1}]	H_2/N_2 [-]	H_2/CH_4 [-]	H_2 permeance [10^{-7} mol m^{-2} s^{-1} Pa^{-1}]	H_2/N_2 [-]	H_2/CH_4 [-]
0	5.2	21	35	2.8	139	1027
90	5.4	17	19	3.4	128	651
150	1.9	23	23	3.8	98	439

References

S1. Sircar, S. Pressure swing adsorption. *Ind. Eng. Chem. Res.* **2002**, *41*, 1389-1392
S2. Adhikari, S.; Fernando, S. Hydrogen membrane separation techniques. *Ind. Eng. Chem. Res.* **2006**, *45*, 875
S3. Bernardo, G.; Araujo, T.; da Silva Lopes, T.; Sousa, J.; Mendes, A. Recent advances in membrane technologies for hydrogen purification. *Int. J. Hydrogen Energ.* **2020**, *45*, 7313-7338
S4. Hinchliffe A. B.; Porter, K. E. A comparison of membrane separation and distillation. *Trans IChemE* **2000**, *78*, 255-268
